# Synaptic Plasticity and Excitation-Inhibition Balance in the Dentate Gyrus: Insights from *In Vivo* Recordings in Neuroligin-1, Neuroligin-2, and Collybistin Knockouts

**DOI:** 10.1155/2018/6015753

**Published:** 2018-02-18

**Authors:** Peter Jedlicka, Julia Muellerleile, Stephan W. Schwarzacher

**Affiliations:** ^1^Institute of Clinical Neuroanatomy, Neuroscience Center, Goethe University Frankfurt, Frankfurt, Germany; ^2^Centre for the 3R-Principle, Faculty of Medicine, Justus Liebig University Giessen, Giessen, Germany

## Abstract

The hippocampal dentate gyrus plays a role in spatial learning and memory and is thought to encode differences between similar environments. The integrity of excitatory and inhibitory transmission and a fine balance between them is essential for efficient processing of information. Therefore, identification and functional characterization of crucial molecular players at excitatory and inhibitory inputs is critical for understanding the dentate gyrus function. In this minireview, we discuss recent studies unraveling molecular mechanisms of excitatory/inhibitory synaptic transmission, long-term synaptic plasticity, and dentate granule cell excitability in the hippocampus of live animals. We focus on the role of three major postsynaptic proteins localized at excitatory (neuroligin-1) and inhibitory synapses (neuroligin-2 and collybistin). *In vivo* recordings of field potentials have the advantage of characterizing the effects of the loss of these proteins on the input-output function of granule cells embedded in a network with intact connectivity. The lack of neuroligin-1 leads to deficient synaptic plasticity and reduced excitation but normal granule cell output, suggesting unaltered excitation-inhibition ratio. In contrast, the lack of neuroligin-2 and collybistin reduces inhibition resulting in a shift towards excitation of the dentate circuitry.

## 1. Introduction

The dentate gyrus is an anatomically and functionally well-characterized region of the mammalian hippocampal formation. Due to its position in the hippocampal circuitry, the dentate gyrus exerts control over the information flow and excitability in the hippocampal formation [1]. Granule cells, the principal neurons of the dentate gyrus, receive their primary excitatory input from stellate cells in the entorhinal cortex, whose axons form the perforant pathway [2]. The perforant path can be divided into two components, one deriving from the more medial portion and one from the more lateral portion of the entorhinal cortex. The medial perforant path carries spatial information and terminates in the middle molecular layer of the dentate gyrus, whereas the lateral perforant path carries sensory information and terminates in the outer molecular layer [3]. It is now well recognized that the dentate gyrus is important for learning and memory, and a variety of dentate-dependent mechanisms have been proposed (for review, see [4]). A common hypothesis for dentate gyrus function is pattern separation, a process that is thought to underlie the ability to distinguish between similar memories because the sparse connectivity between granule cells and CA3 pyramidal cells ensures that no two pyramidal cells receive input from the same subset of granule cells [5, 6] (for recent reviews, see [7, 8]). Individual granule cells may also be involved in spatial recognition by functioning as place cells, which are only active when an animal is in a specific “place field” [9–11]. Due to the convergence of spatial and nonspatial information in the dentate gyrus, it is hypothesized that the dentate may also encode contextual memories by integrating spatial information with sensory information in a process called conjunctive encoding or binding [3, 12].

Perforant-path stimulation is a classical *in vivo* model to study synaptic transmission, long-term synaptic plasticity, and network excitability in the dentate gyrus [13]. *In vivo* recordings have the advantage that the intrinsic connectivity, excitation/inhibition (E/I) balance, and network activity are preserved, in contrast to other experimental manipulations where these may be compromised. Some types of organotypic hippocampal slice cultures may exhibit alterations in E/I balance due to the development of aberrant connections leading to increased dentate granule cell excitability [14–16] (but see also [17]). Granule cells in acute hippocampal slices may exhibit a lower amount of inhibition than *in vivo* because some interneuronal axons and dendrites are cut off during the slicing procedure [18]. Furthermore, maintaining the proper oxygen level for submerged slices is crucial for upholding naturalistic network activity *in vitro* [19]. Due to the low firing frequency and difficulty of targeting individual granule cells in awake animals [20] and also due to the difficulty in isolating granule cell single units (because cells are tightly packed and generally quiet), perforant path stimulation and recording of the population activity of granule cells in anesthetized animals is a useful method for studying network activity *in vivo*. Most *in vivo* electrophysiological studies of the dentate gyrus have been carried out in rats. However, during the last years, interest in studying synaptic transmission and plasticity in the mouse *in vivo* has arisen since transgenic or conventional and conditional knockout mice provide the opportunity to examine the function of novel genes and proteins.

Synaptic plasticity is a process in which synaptic inputs change their strength as a consequence of their previous activation. Long-term potentiation (LTP), a long-lasting increase in synaptic efficacy, is widely accepted as a basic mechanism for learning and memory. Besides learning and memory, long-term synaptic plasticity plays a crucial role in a variety of both physiological and pathophysiological conditions including epilepsy, addiction, neurodegenerative diseases, or mechanisms of pain [21]. Generally, high-frequency afferent activity leads to calcium influx through NMDA receptors and thereby causes an increase of intracellular calcium concentration which mediates the induction of LTP [22]. The mechanism for the expression of LTP mainly depends on changes in the number and/or properties of AMPA receptors in the postsynaptic membrane. Additional AMPA receptors are inserted into the postsynaptic membrane after induction of LTP [23]. LTP can be effectively induced in granule cells *in vivo* by high-frequency stimulation of presynaptic perforant path fibers [13, 24–26].

**Figure 1:**
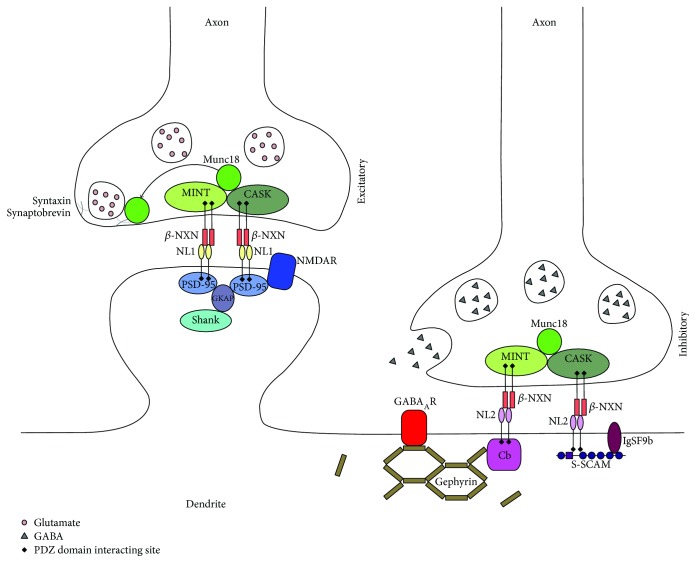
Diagram depicting the localization and interactions of NL1, NL2, and Cb at excitatory and inhibitory synapses. The extracellular domains of NL1 and NL2 bind presynaptic neurexins, which are bound to the presynaptic scaffold proteins Munc18 interacting protein (MINT) and calcium/calmodulin-dependent serine protein kinase (CASK). NL1 is located at excitatory synapses and binds the postsynaptic density protein 95 (PSD-95) intracellularly, which also binds NMDA receptors. NL1 also directly or indirectly binds the Shank family scaffolding proteins. NL2 is located at inhibitory synapses and binds to synaptic scaffolding molecule (S-SCAM) as well as collybistin, which recruits the scaffolding protein gephyrin from intracellular stores (not shown) to the postsynapse. Gephyrin mediates the accumulation of GABA_A_ receptors to the membrane (for reviews, see [32, 84]).

In the dentate gyrus, glutamatergic and GABAergic synapses act as the major sources of excitation and inhibition, respectively [2, 27]. Incoming glutamatergic perforant path fibers form excitatory synapses on both the principal glutamatergic granule cells and neighboring GABAergic interneurons, for example, basket cells [28]. Activated basket cells thus provide direct feed-forward inhibition via somatic GABAergic synapses on granule cells, and, following glutamatergic excitation through granule cells, together with hilar interneurons exert additional strong dendritic and somatic feed-back inhibition and thus regulate the excitatory output of granule cells to the CA3 [27, 29–31]. Together, these mechanisms form a local network to balance excitation and inhibition, which is crucial for efficient information processing. Fast excitatory/inhibitory transmission is predominantly mediated by ionotropic AMPA/GABA_A_ receptors. The functional integrity of fast excitatory and inhibitory transmission is essential for normal neuronal activity. Therefore, it is important to study the delicate control mechanisms which exist in neurons to regulate the function of excitatory and inhibitory synapses. Little is known about the ways in which these mechanisms shape the synaptic properties and the output of dentate granule cells. Identification and functional characterization of crucial molecular players at glutamatergic and GABAergic inputs to granule cells is critical for understanding the role of the dentate gyrus in learning and memory. In this minireview, we focus on a major question: What are the effects of key molecular regulators of excitatory/inhibitory synaptic transmission on long-term synaptic plasticity and granule cell excitability in an intact dentate gyrus circuitry of live animals? We summarize studies of knockout (KO) mice shedding light on the role of three proteins (neuroligin-1, neuroligin-2, and collybistin) involved in the regulation of glutamatergic and GABAergic transmission (Figure 1).

## 2. Neuroligin-1 Regulates Excitatory Synaptic Transmission and LTP in the Dentate Gyrus

Neuroligins are transmembrane cell adhesion proteins that are involved in the regulation of excitatory and inhibitory synapses [32, 33]. Neuroligins are clinically highly relevant since disruption of their function has been proposed to contribute to neuropsychiatric abnormalities such as learning deficits and autism [34]. Studies using neuronal cultures and acute slices from different brain areas and in different species support a selective role for neuroligin-1 (NL1) in the function of glutamatergic synapses [35]. Chen et al. [36] found that the knockdown of NL1 in developing neurons in the optic tectum of *Xenopus laevis* tadpoles led to a decrease in synapse densities and AMPA receptor current frequency. Dissociated hippocampal cultures from newborn NL1 KO mice (KO) also exhibited a decrease in AMPA receptor current frequency and amplitude as well as AMPA receptor cluster density [37], but other research points to a specific role for NL1 in regulating NMDA receptor-dependent transmission in mature neurons [38]. Another *in vitro* study showed that NL1 is phosphorylated by CaMKII, a key regulator of activity-dependent synaptic plasticity, and that the surface expression of NL1 is increased following increased network activity in cortical cultures [39]. Further supporting a role for NL1 in synaptic plasticity, the knockdown of NL1 decreases perforant path-granule cell LTP in acute slices of the adult rat dentate gyrus but affects LTP in the CA1 of young rats only [40]. A different study using a conditional knockout approach showed that the selective loss of NL1 in CA1 pyramidal neurons in mice abolished both NMDA receptor-dependent and receptor-independent LTP in acute slices [41]. The apparent inconsistency with the results from [40] may be explained by the difference in methods; a conditional genetic knockout leads to a complete loss of the target protein whereas a microRNA-mediated knockdown may not eliminate all protein. Based on this previous research on the topic, it can be concluded that NL1 plays a role in excitatory synaptic transmission and LTP in the hippocampal formation and in other brain areas.

**Figure 2:**
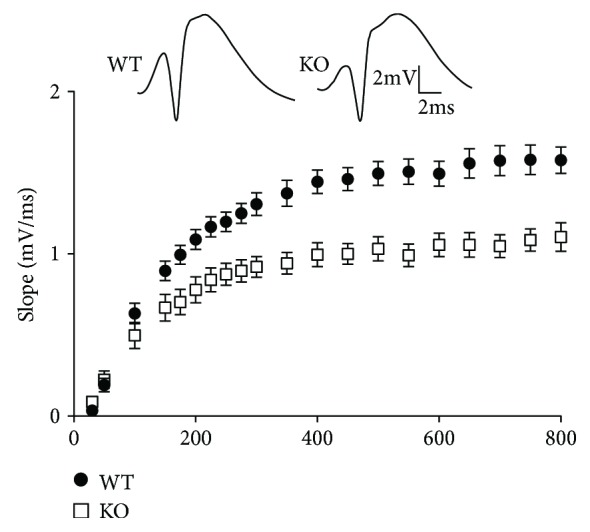
The lack of neuroligin-1 (NL1) leads to impaired synaptic transmission at excitatory perforant path synapses. This was shown by impaired stimulus-response relationship for the slope of the field excitatory postsynaptic potential (fEPSP) in NL1 knockout (KO) mice. The fEPSP slope is a measure for the strength of synaptic transmission. Field potential responses were recorded in the hilus of the dentate gyrus. Slopes of fEPSPs were decreased in NL1 KO mice relative to their wild-type (WT) littermates. Top: representative recordings from one WT and one NL1 KO animal at 500 *μ*A stimulus strength (adapted with permission from [42]).

In line with these observations, perforant path stimulation and field potential recordings of granule cells *in vivo* in urethane-anesthetized NL1 KO mice revealed strongly reduced synaptic responses upon the activation of glutamatergic perforant path granule cell inputs (Figure 2). In addition, NL1 KO mice showed significantly reduced expression levels of NMDA receptor subunits GluN1, GluN2A, GluN2B and the AMPA receptor subunit GluA2 in synaptosomal hippocampal preparations [42]. This reduction in glutamatergic receptors is consistent with the impaired excitatory transmission found in NL1 KOs *in vivo*. The reason for diminished excitatory responses might be loss of synapses or loss of receptors within synapses. Shipman and Nicoll [40] found that knockdown of NL1 reduced the number of synaptic inputs rather than the number of NMDA/AMPA receptors per synapse (see also [43]). In contrast, analyses of CA1 area in NL1 KOs have revealed changes in the AMPA/NMDA ratio. Further studies are needed to nail down the precise mechanism of reduced excitatory responses in the NL1-deficient dentate gyrus.

Because of the reduced NMDA receptor levels [42], reported reduction in AMPA/NMDA ratio in NL1 KOs [44] and observed involvement of NL1 in the recruitment of NMDARs [45], it was hypothesized that NL1 might be involved in the regulation of long-term synaptic plasticity in the dentate gyrus. Indeed, *in vivo* recordings demonstrated that NL1 KOs displayed diminished LTP [42], in keeping with the previously reported reduced excitation and LTP upon microRNA-mediated knockdown of NL1 in the dentate gyrus [40]. Moreover, in agreement with deficits in synaptic plasticity, mice lacking NL1 displayed impairments in spatial memory [44]. In line with this, NL1 KOs showed impaired LTP in the CA1 area of the hippocampus associated with reduced NMDA/AMPA ratio [44]. However, although NL1 deletion caused LTP deficits both in the CA1 [44] and the dentate gyrus [40, 42], this does not mean that the underlying molecular mechanisms are the same. Indeed, molecular mechanisms of LTP at perforant path synapses are partially similar [46] but not entirely identical with the mechanisms at Schaffer collateral-CA1 synapses. For example, the autophosphorylation of CAMKII is required in the CA1 but not in the dentate gyrus [47, 48]. It also cannot be excluded that, although LTP was impaired upon the loss of NL1 [40, 42, 44], NL1 involvement in LTP regulation may be indirect, that is, permissive rather than instructive (see the discussion of LTP in [49], but see also [37, 41, 42, 45]). Nevertheless, LTP as well as input-output data suggest that, by modulating these granule cell features, NL1 might contribute to dentate gyrus-related mnemonic functions, such as spatial pattern separation [4] or binding of sensory and spatial information [12]. It would be interesting to test this hypothesis in future studies.

Of note, while the medial and lateral perforant path both exhibit NMDA receptor-dependent plasticity that is affected by noradrenergic signaling [50], there also are differences regarding their plasticity mechanisms. For instance, LTP at lateral perforant path-granule cell synapses requires the activation of opioid receptors [51]; and endocannabinoids mediate lateral perforant path LTP [52], but suppress glutamate release from medial perforant path synapses [53]. Field excitatory postsynaptic potentials (fEPSPs) induced by lateral and medial perforant path stimulation in acute slices also differ in their kinetics, suggesting differences in the vesicle release probabilities of these synapses [54, 55]. Therefore, it is important to note which pathway is being studied. In all *in vivo* studies reviewed in this article, medial perforant path synaptic responses and their plasticity were investigated. Thus, future work is needed to test whether the conclusions about the role of NL1 (see above), NL2, and Cb (see below) at perforant path synapses will hold also for lateral perforant path synapses.

**Figure 3:**
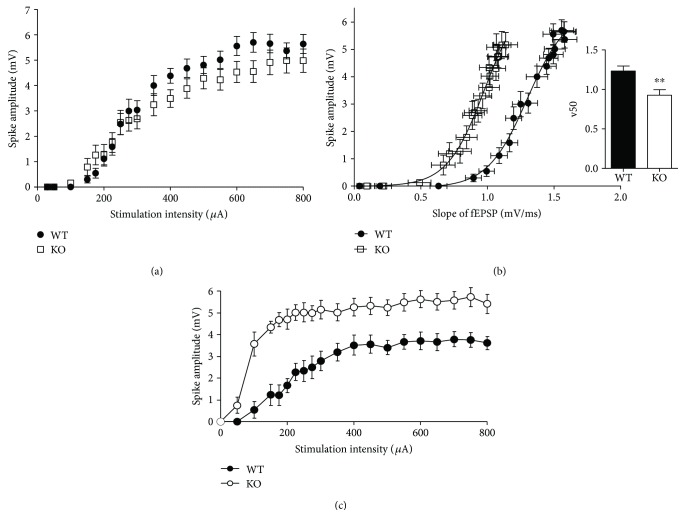
The influence of NL1 and NL2 on synaptic transmission and granule cell firing. (a) Normal granule cell firing in the NL1-deficient dentate gyrus. Population spike is a measure of granule cell firing. Input-output relationship for population spike amplitudes recorded in WT and NL1 KO mice was not changed indicating similar capability for generating action potentials. (b) EPSP-spike (ES) analysis revealed enhanced coupling between the slope of the fEPSP and the amplitude of the corresponding population spike evoked by perforant path stimulation. Inset: a significant (unpaired *t*-test, ^∗∗^*p* < 0.01) decrease in the slope generating 50% of maximal spike amplitude (v50) was found in NL1 KO as compared to WT animals (adapted from [42]). (c) The lack of NL2 leads to a dramatic increase in granule cell excitability. Increased amplitude of population spikes in the absence of NL2 implies higher number and synchrony of firing in NL2-deficient granule cells following perforant path stimulation (adapted with permission from [42, 59]).

Intriguingly, although in the *in vivo* recordings granule cells displayed an impairment of their input excitation, their output in the form of action potential firing was not compromised in the absence of NL1 (Figure 3(a)) [42]. This was due to a greater EPSP-spike (E-S) coupling in NL1 KO than in WT mice (Figure 3(b)). Furthermore, paired-pulse inhibition of the granule cell population spike was reduced in NL1 KOs, which indicated weaker GABAergic network inhibition. Thus, the increase in the responsiveness of granule cells to EPSPs seems to result from decreased GABAergic inhibition. The reason for this might be that NL1 deletion most likely affects excitatory perforant path inputs not only to granule cells but also to inhibitory interneurons. Viewed together, data from NL1 KO animals have shown that NL1 plays a prominent role in the regulation of excitatory transmission and LTP in the hippocampal dentate gyrus.

It may be of clinical relevance that NL1 KO mice exhibit enhanced repetitive (stereotypic) behaviors, which are key symptoms of autism and have been attributed to impairments of corticostriatal synaptic transmission [44]. Behavioral and functional changes observed in these mutants as well as other recently generated KO mice [56, 57] corroborate current working hypotheses that deficits in glutamatergic synaptic transmission caused by the loss of proteins at excitatory synaptic inputs may underlie neurological deficits in patients suffering from autism spectrum disorders. This does not mean that changes in the dentate gyrus are directly implicated in the pathophysiology of autism. However, a better understanding of the role of NL1 in shaping E/I balance in the dentate gyrus might lead to some insights into its role in other brain regions directly involved in the pathological phenotype.

## 3. Neuroligin-2 Regulates Perisomatic Inhibitory Synaptic Transmission in the Dentate Gyrus

**Figure 4:**
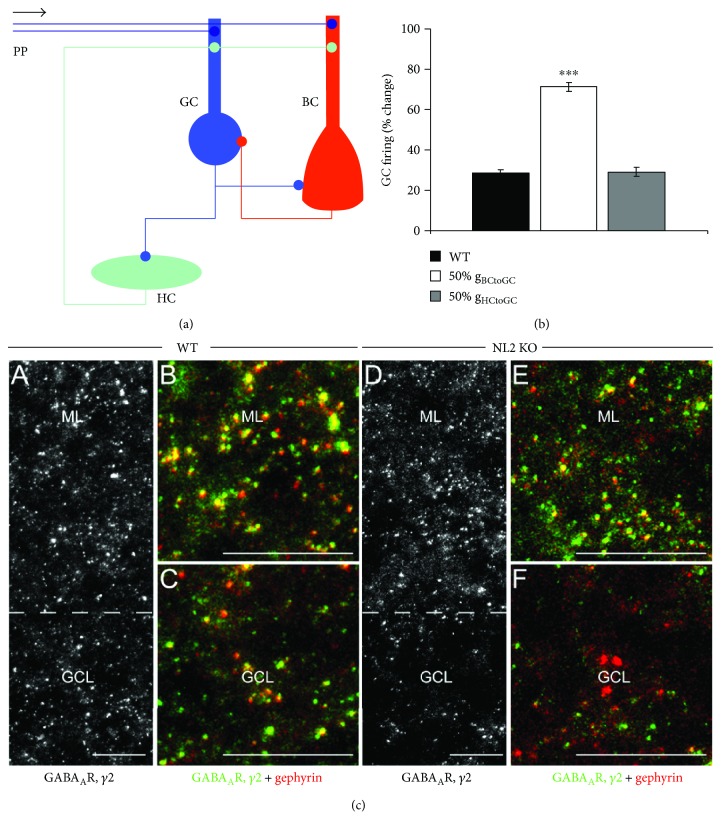
Biologically detailed network modelling predicted that reduced paired-pulse inhibition of granule cell firing observed in NL2 KO mice was due to the reduction of somatic GABAergic inhibition mediated by basket cell synapses. (a) Schematic circuit depicting connections among excitatory granule cells (GC) and inhibitory basket cells (BC) and hilar cells (HC) in the network model [62]; mossy cells (MC) not shown). (b) Quantification of simulation data on network inhibition of granule cell firing. Network inhibition is weaker and GC firing is significantly higher (*t*-test, ^∗∗∗^*p* < 0.001) in the simulated NL2 KO network model with reduced GABA_A_ conductance (50% reduction of maximum synaptic conductance) at somatic (BC-to-GC) inhibitory synapses (g_BCtoGC_). Note that no significant impairment of GC network inhibition was observed in the network model with a selective reduction (50%) of GABA_A_ synaptic conductances at dendritic HC-GC synapses (g_HCtoGC_). (c) The punctate immunostaining of sections from WT (A-C) and NL2 KO mice (D–F) for *γ*2-subunit of GABA_A_ receptors and inhibitory postsynaptic marker gephyrin. Modelling predicted and experiments confirmed that diminished network inhibition of GC firing, found in electrophysiological recordings in NL2 KO animals, was accompanied by a reduction of somatic GABA_A_ receptor clusters in the granule cell layer (GCL) of the dentate gyrus. Note that the number of GABA_A_ receptor clusters located in the dendritic molecular layer (ML) of the dentate gyrus was not changed in NL2 KO animals. Also, the colocalization of GABA_A_ receptor *γ*2-subunit and gephyrin was selectively reduced in the GCL but not in the ML of NL2 KO mice (adapted with permission from [59]).

Neuroligin-2 (NL2) is a postsynaptic adhesion protein which is present at inhibitory synapses [58]. In contrast to NL1 KO mice, field potential recordings in the dentate gyrus of NL2 KO mice [59] revealed strongly enhanced granule cell firing (Figure 3(c)). Patch-clamp experiments in acute hippocampal slices detected reduced GABA_A_ receptor-mediated miniature inhibitory postsynaptic currents in granule cells of NL2 KO mice as compared to WT controls [59] (see also [60, 61]). In line with this, paired-pulse inhibition of the population spike was strongly reduced in NL2 KOs thus confirming *in vivo* the disruption of GABAergic network inhibition upon deletion of NL2 [59]. In order to analyze these findings at the level of neuronal circuit, an established detailed network model [62] was used to simulate granule cell activity [29, 63] observed in extracellular field recordings. The model was an anatomically and physiologically realistic network model of the dentate gyrus (Figure 4(a)) comprised of multicompartmental neuron models representing four major excitatory and inhibitory cell types (granule cells, mossy cells, basket cells, and hilar cells), reproducing their electrophysiology and firing behavior [62].

Computer simulations in this network model predicted that impaired paired-pulse inhibition of granule cell firing (a measure of network inhibition of granule cell firing) observed in NL2 KO mice is mainly caused by diminished perisomatic inhibition of granule cells (Figure 4(b), see also [29]). Consistent with this computational prediction, immunohistological analyses revealed significantly decreased numbers of GABA_A_ receptor and gephyrin clusters in the granule cell layer of NL2 KOs, indicating a loss of synaptic GABA_A_ receptors from the somata of granule cells. Importantly, similar changes of somatic GABA_A_ receptor and gephyrin clusters accompanied by reduced inhibitory currents have been observed in CA1 pyramidal cells in NL2 KOs [60].

These data indicate that NL2 is a key regulator of perisomatic GABAergic inhibition in the hippocampal formation. Interestingly, it seems that NL2 plays a similar synapse-specific role also in other areas of the brain such as amygdala [61]. In agreement with this, an *in vitro* patch-clamp study in the neocortex has revealed that NL2 deletion selectively decreases inhibitory synaptic currents originating from interneurons mediating perisomatic but not dendritic inhibition [64]. Thus, although NL2 is present at dendritic as well as somatic inhibitory inputs, only somatic inhibitory transmission seems to be dependent on NL2. Taken together, electrophysiological and immunohistological data combined with computational modeling demonstrated that the lack of NL2 impaired GABAergic inhibition and increased excitability of granule cells in the dentate gyrus of live animals. Interestingly, an NL2 nonsense variant has recently been reported in a human patient for the first time and was associated with autism, anxiety, and intellectual impairment [65]. This suggests that alterations in excitation-inhibition balance due to loss of NL2 in brain regions involved in autism-related behavior and anxiety may lead to severe cognitive deficits.

What are the molecular mechanisms underlying the synapse- and location-specific effects of NL2 action? Why does NL2 deletion impair only somatic inhibition without affecting dendritic inhibition? NL2 contributes to the clustering of perisomatic GABA_A_ receptors via its interaction with gephyrin and activation of collybistin [60, 66] (see also below). Recent studies suggest that complex interactions of NL2 with other synaptic adhesion proteins such as MDGA1 [67, 68] and IgSF9b [69] may contribute to its location-specific role in the regulation of neuronal inhibition [70].

## 4. Collybistin Regulates Inhibitory Synaptic Transmission and Modulates LTP in the Dentate Gyrus

Collybistin (Cb) is a brain-specific guanine nucleotide exchange factor, which interacts with the synaptic scaffolding protein gephyrin [71]. Collybistin and gephyrin are important for the clustering of GABA_A_ receptors at inhibitory postsynapses [72, 73].

**Figure 5:**
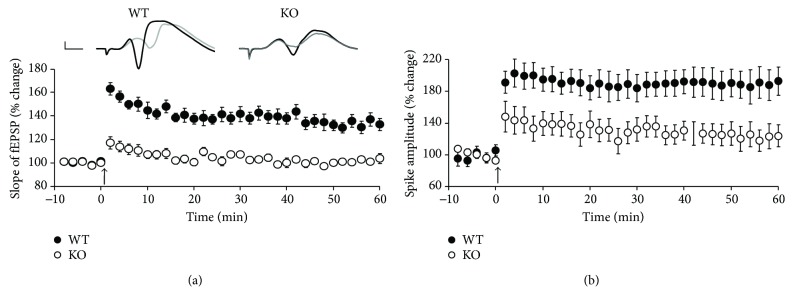
Reduced LTP in the dentate gyrus of collybistin (Cb) KO mice as compared to WT littermates. Mean fEPSP slope (a) and population spike (b) changes are plotted as a function of time. Arrows denote the start of the theta-burst stimulation protocol consisting of 6 series of 6 trains of 6 pulses at 400 Hz with 200 ms between trains and 20 s between series. Top in (a): fEPSPs recorded before (grey) and after the induction of LTP (black). Calibration bars: 1 mV, 2 ms (adapted with permission from [74]).

*In vivo*, the Cb-deficient dentate network exhibited a significantly lower threshold for the population spike demonstrating enhanced excitability of granule cells [74]. In line with this, the number of postsynaptic gephyrin and GABA_A_ receptor clusters was significantly smaller in Cb KO animals. These results are consistent with *in vitro* findings in area CA1, which demonstrate reduced dendritic inhibition following Cb deletion [75]. Interestingly, LTP was impaired (Figure 5) in Cb KO mice. The reduction of long-term plasticity was most likely mediated by decreased inhibition and subsequent prepotentiation of synaptic transmission, which saturated LTP and prevented further potentiation. This was supported by the observation of steeper fEPSP slopes in input-output curves of Cb KO mice as compared to WT mice. The same effect of decreased inhibition on LTP was shown in a recent *in vivo* study where the knockdown of the receptor tyrosine kinase EphA7, which is implicated in gephyrin clustering, led to a specific reduction in perisomatic basket cell-granule cell synapses and decreased LTP at perforant path-granule cell synapses [76]. Therefore, similarly to NL2, Cb is required for normal GABAergic inhibition and excitation/inhibition balance in the hippocampus *in vivo*. Importantly, the finding that functional deficits in the dentate gyrus of Cb KO mice were associated with a significant reduction of synaptic gephyrin and GABA_A_ receptor clusters indicates that Cb is an important determinant of gephyrin-dependent GABAergic mechanisms of network excitability. Recent *in vitro* studies have revealed further molecular details of Cb activation including GTPase-dependent signaling [77] as well as binding of Cb to NL2 [66] and NL2-dependent binding of Cb to phosphatidylinositol 3-phosphate [66, 78, 79], contributing to the recruitment of gephyrin and GABA_A_ receptors to inhibitory synapses.

## 5. Summary

**Figure 6:**
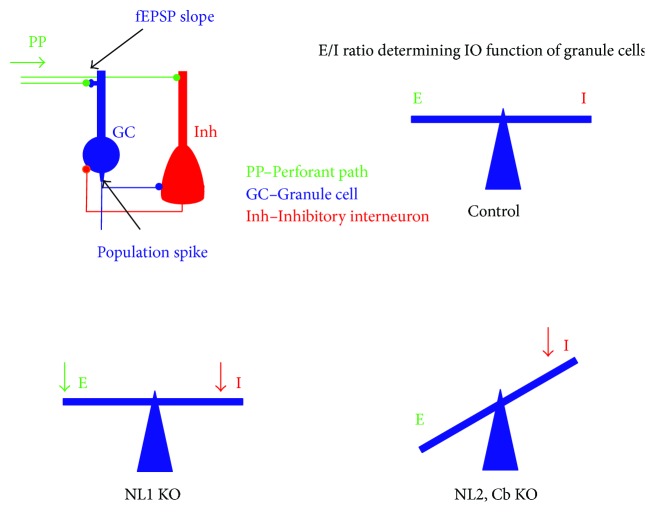
Summary of the effects of NL1, NL2, and Cb deletion on E/I ratio and granule cell I/O function. Whereas deletion of NL1 does not change output firing of granule cells, deletion of NL2 and Cb leads to enhanced granule cell output (see text for details).

We have reviewed the role of three major molecular players (NL1, NL2, and Cb) in the regulation of glutamatergic excitation and GABAergic inhibition in dentate granule cell function *in vivo*. We have provided an overview of several studies, which used *in vivo* electrophysiology, immunohistochemistry, and computational modelling to examine how the lack of these molecules affects synaptic properties and neuronal activity in the dentate gyrus circuit. Findings from these studies demonstrate that whereas NL1 is required for physiological levels of synaptic plasticity at glutamatergic perforant path-granule cell synapses, NL2 and Cb are critical for normal function of GABAergic inhibitory synapses in the dentate gyrus *in vivo*. *In vivo* recordings of population spikes allow for the assessment of the effects of the lack of these molecules on the input-output (I/O) function of granule cells embedded in a dentate circuitry with intact connectivity. I/O firing properties of dentate granule cells are mainly determined by E/I ratio and intrinsic cellular properties. *In vivo* electrophysiology experiments and computational modelling indicated that strongly diminished inhibition upon deletion of NL2 and Cb led to an increase in the E/I ratio and enhanced granule cell output (Figure 6). In contrast, deletion of NL1 reduced not only the excitation of granule cells but also most likely the excitation of inhibitory interneurons and thereby did not disrupt the overall E/I ratio. This is supported by the observation of unaltered granule cell firing in the absence of NL1.

The studies described in this minireview may provide insights into synaptic mechanisms of information processing in the dentate network. Importantly, neuroligins and Cb have been proposed to play a role in the development of autism, learning deficits, and seizures [80, 81]. In addition, disruption of E/I balance is considered to underlie neurological deficits in autism and schizophrenia (e.g., [82, 83]). Findings from the dentate gyrus might contribute to uncovering general principles for molecular regulation of synaptic E/I balance and neuronal excitability. Although the dentate gyrus is not directly involved in symptoms of schizophrenia or autism, general insights on regulation of E/I balance may help better understand the pathogenesis of these neurological disorders. Moreover, pathological changes in the dentate gyrus play a crucial role in temporal lobe epilepsy [1], and therefore, *in vivo* studies of dentate gyrus excitability and plasticity facilitate the search for mechanisms of epileptogenesis.
